# PROMOTING THE PATIENT EXPERIENCE AND WELL-BEING: LESSONS FROM GROUNDBREAKING PROJECTS IN HEALTH CARE DESIGN

**DOI:** 10.1093/geroni/igae098.2025

**Published:** 2024-12-31

**Authors:** Elisabeth Perreault, Margi Kaminski

**Affiliations:** CannonDesign, Buffalo, New York, United States; CannonDesign, Chicago, Illinois, United States

## Abstract

The speakers will discuss how the built environment can and should support older adults in healthcare, rehabilitation, and long-term care facilities. They will share two case studies that showcase design solutions for older adults informed by the Living-Centered Design approach and focusing on long-term care and memory care for veterans, and recovery and rehabilitation, respectively. They will discuss current best practices in healthcare and long-term care design for older adults, and how the built environment can actively assist patient recovery and well-being. The first case study is the Veterans Home of California-Yountville (VHC-Yountville), which is under construction and nearing completion. This is a new 240-bed and 285,000 square feet (SF) skilled nursing and memory care facility by the California Department of Veterans Affairs (CalVet) and is located on CalVet’s historic campus in Napa Valley. Evidence-based design principles for older adults, including those with dementia and traumatic brain injuries, were applied to create a supportive, long-term care environment that effectively balances medical needs with the need for comfort and community. The second case study located in the state of New York is a new 9-story, 168-bed, and 189,600 SF urban rehabilitation facility for older adults focusing on patient-directed care. The patient services provided include medical care, physical, occupational, and speech therapy, diagnostic/ lab services, palliative care, social work, and mental health services. “Greenhouse” design principles were applied to create a post-acute rehabilitation facility that showcases how the building can be designed to actively catalyze and support patient recovery and rehabilitation.

